# Forces and moments generated by 3D direct printed clear aligners of varying labial and lingual thicknesses during lingual movement of maxillary central incisor: an in vitro study

**DOI:** 10.1186/s40510-023-00475-2

**Published:** 2023-07-10

**Authors:** James Grant, Patrick Foley, Brent Bankhead, Gabriel Miranda, Samar M. Adel, Ki Beom Kim

**Affiliations:** 1 Department of Orthodontics, Saint Louis University, 3320 Rutger St, St Louis, MO 63104 USA; 2 Department of Orthodontics, Faculty of Dentistry, Alexandria University, Alexandria, Egypt

**Keywords:** Direct printed aligners, Digital orthodontics, Forces, Moments

## Abstract

**Objective:**

The objective of this study was to measure the forces and moments exerted by direct printed aligners (DPAs) with varying facial and lingual aligner surface thicknesses, in all three planes of space, during lingual movement of a maxillary central incisor.

**Materials and methods:**

An in vitro experimental setup was used to quantify forces and moments experienced by a programmed tooth to be moved and by adjacent anchor teeth, during lingual movement of a maxillary central incisor. DPAs were directly 3D-printed with Tera Harz TC-85 (Graphy Inc., Seoul, South Korea) clear photocurable resin in 100-µm layers. Three multi-axis sensors were used to measure the moments and forces generated by 0.50 mm thick DPAs modified with labial and lingual surface thicknesses of 1.00 mm in selective locations. The sensors were connected to three maxillary incisors (the upper left central, the upper right central, and the upper left lateral incisors) during 0.50 mm of programmed lingual bodily movement of the upper left central incisor. Moment-to-force ratios were calculated for all three incisors. Aligners were benchtop tested in a temperature-controlled chamber at intra-oral temperature to simulate intra-oral conditions.

**Results:**

The results showed that increased facial thickness of DPAs slightly reduced force levels on the upper left central incisor compared to DPAs of uniform thickness of 0.50 mm. Additionally, increasing the lingual thickness of adjacent teeth reduced force and moment side effects on the adjacent teeth. DPAs can produce moment-to-force ratios indicative of controlled tipping.

**Conclusions:**

Targeted increases in thickness of direct 3D-printed aligners change the magnitude of forces and moments generated, albeit in complex patterns that are difficult to predict. The ability to vary labiolingual thicknesses of DPAs is promising to optimize the prescribed orthodontic movements while minimizing unwanted tooth movements, thereby increasing the predictability of tooth movements.

## Introduction

Over the past few decades, the number of adult patients seeking orthodontic treatment has increased. This can be attributed to the profession’s ability to treat problems effectively and patients’ desire to maintain their natural teeth and improve their appearance. Advances in materials science and CAD-CAM technologies, including optical intra-oral scanning and 3D printing, have led to a surge in the popularity of clear aligners as a treatment modality in orthodontics. Clear aligners gradually align teeth with successive stages of aligners toward their virtually pre-programmed ideal alignment. The superior esthetics of clear aligners over fixed appliances and significant corporate marketing have contributed to their widespread adoption [1–3].

The conventional method of fabricating clear aligners involves multiple steps, including obtaining a dental impression or intra-oral scanning, producing a dental model, thermoforming a biocompatible plastic to the model, and trimming and polishing the margins. This process becomes time-consuming when numerous aligners are required [4].

Moreover, conventional thermoformed aligners have limitations such as significant geometric inaccuracies, dimensional instability, low strength, and reduced wear resistance. These problems can be attributed to the materials used or the thermoforming process itself. Furthermore, some clear aligner materials exhibit narrow ranges for molding temperature beyond which the aligner material oxidizes, diminishing the aligner transparency and esthetics [5–8].

Apart from the aligner material, aligner thickness is one of the most influential factors affecting the biomechanics of clear aligners and hence the force magnitude delivered to the teeth [8, 9]. Several studies demonstrate that thicker aligners generate greater force delivery [10–13]. However, thermoformed aligners do not have uniform thickness; they are thicker in the posterior than the anterior regions, and the thickness is generally reduced following the thermoforming process [14]. This is a major limitation of thermoformed aligners since thickness is a key property that affects its force profile and load–deflection. Non-planned variations in aligner thickness pose a challenge to accurately execute programmed tooth movements because aligner thickness is a major determinant of force delivery and its stress-relaxation rate. Indeed, Invisalign® (Align Technology, Santa Clara, CA), the first and largest mass-marketed solution for clear aligner therapy currently on the market, reports an overall pre-programmed tooth movement accuracy of only 46–56% [15].

Interestingly, conventional thermoformed clear aligners were found to generate force levels three to eleven times higher than ideal force ranges during maxillary incisor tipping [12]. On the contrary, directly 3D-printed aligners (DPAs) generate more consistent and lower forces than their thermoformed counterparts that are far closer to the ideal range stated by Proffit [16].

With wider adoption of in-house treatment planning software, intra-oral scanners, and 3D printers, clinicians are increasingly adopting in-house aligner solutions [17–19]. The ability to directly 3D-print the clear aligners eliminate many intermediate fabrication steps, reducing labor time and waste. In addition, improved dimensional accuracy and more ideal force ranges for tooth movement can be achieved.

The purpose of the present study was to investigate the forces and moments generated by DPAs during in vitro lingual movement of a maxillary central incisor, when the aligners’ labial or lingual thicknesses were selectively increased in the regions around the site of programmed tooth movement. The central incisor was selected to study tooth movement by 3D direct printed clear aligners as it is located in the middle of the dentition and patients are always concerned about the esthetic improvement of anterior teeth.

## Materials and methods

### Experimental apparatus

**Fig. 1:**
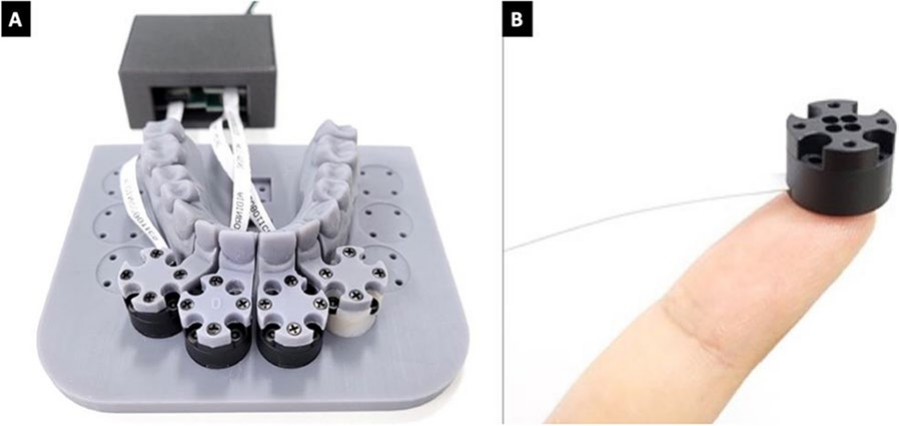
Experimental apparatus of multi-axis force/moment sensors. **A** The experimental apparatus with three multi-axis force/moment transducers connected to the upper central incisors and the upper left lateral incisors. **B** An individual AFT20-D15 multi-axis force/moment transducer

A benchtop hardware/software setup was used to measure the moments and forces generated by DPAs on the upper right central incisor (UR1) and the upper left central and lateral incisors (UL1, UL2). The hardware setup consisted of a 3D-printed maxillary arch with a modular design, where all four incisors were individually 3D-printed and attached to distinct multi-axis force/moment transducers. Three AFT20-D15 multi-axis force/moment transducers (Aidin Robotics, Anyang, South Korea) were mounted on a 3D-printed baseplate that was attached to the 3D-printed UR1, UL1, and UL2. The upper right lateral incisor and posterior maxillary arch segments were mounted separately (Fig. 1). The UL1 was printed with a baseline position that was displaced 0.50 mm facially in the hardware setup to simulate the ideal alignment through lingual movement. The experimental apparatus was placed in a semi-enclosed chamber that maintained a constant temperature of 37 °C, simulating the intra-oral conditions.

### Direct print aligner fabrication

**Fig. 2:**
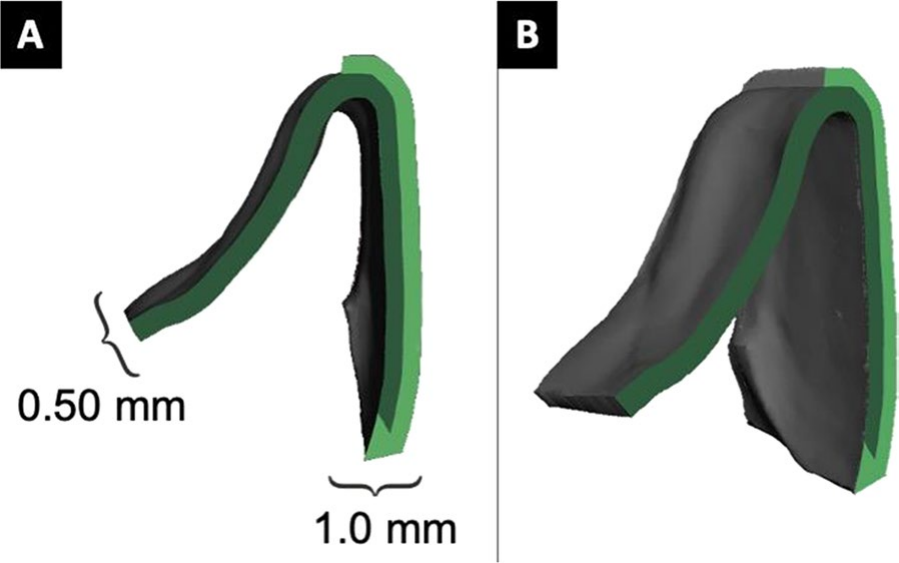
Cross-sections demonstrating increased labial thickness of aligner. **A** Orthogonal view with accompanying thicknesses depicted. **B** Oblique view

**Fig. 3:**
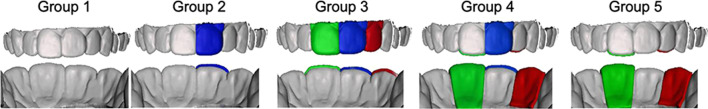
Diagram of the varying labiolingual thicknesses by the five experimental groups

The fabrication process for direct print aligners (DPAs) involved the creation of fifty aligners, which were divided into five experimental groups, each containing ten replicates of active aligners. The active aligners were programmed with 0.50 mm of bodily palatal translation of the upper left central incisor to restore ideal alignment of the maxillary dentition hardware setup. To create varying labial and lingual thicknesses in specific patterns across the UR1, UL1, and UL2 teeth, DPAs were designed for each experimental group, with a baseline aligner of uniform thickness of 0.50 mm (Fig. 2). Group 1 had an overall uniform aligner thickness of 0.50 mm and served as the control to Groups 2–5. Group 2 had an increased cross-sectional thickness of 1.0 mm on the facial surface of UL1. Group 3 had an increased cross-sectional thickness of 1.0 mm on the facial surfaces of UR1, UL1, and UL2 teeth. Group 4 had an increased cross-sectional thickness on the facial surface of UL1 and the palatal surfaces of UR1 and UL2. Group 5 had an increased cross-sectional thickness on the palatal surfaces of UR1 and UL2 (Fig. 3).

To plan the lingual bodily displacement of the UL1 by 0.50 mm and digitally generate the stereolithography (STL) files for the DPAs, a custom version of uDesign 6.0 digital treatment planning software (uLab Systems, Memphis, TN) was used. These DPAs were designed with trim heights flush with the free gingival margins and 0.05 mm offset from the tooth surfaces before being exported in both 0.50 mm and 1.00 mm thickness STL files. The corresponding 0.50 mm and 1.00 mm thick STL files were then superimposed in MeshMixer 3.5 (Autodesk Inc., Mill Valley, CA), and the Plane Cut tool was used to section the 1.00 mm DPA mesh file to leave a 1.00 mm thick labial or lingual surface overlayed on the 0.50 mm thick DPA mesh file, dictated by the patterns of experimental Groups 2–5. The Combine tool was then used to merge the base 0.50 mm and sectioned 1.00 mm thick STL meshes and exported as a single STL mesh file. The DPA files were then imported into UnizMaker (Uniz Technology, San Diego, CA) to prepare for 3D printing.

The occlusal plane of each aligner was rotated 70º from the build platform, and supports were added to the cameo surface. The aligners were printed using SprintRay Pro 95 (SprintRay, Los Angeles, CA) and TC-85 clear photocurable resin (Graphy Inc., Seoul, South Korea) in 100-µm layers. After printing, the aligners were separated from the build platform, and residual resin was removed using a centrifuge. Then, the supports were removed from the aligners, and the aligners were cured in a nitrogen chamber (Tera Harz Cure, Graphy Inc., Seoul, South Korea) for 14 min.

### Data collection

The DPAs were stored in airtight foil bags after fabrication until measurements were taken. Prior to measurements, the test apparatus was heated to a constant 37 °C, and a water bath was heated to 69.4 °C, which corresponds to the glass transition point of TC-85 resin. Each aligner was submerged in the water bath for five seconds, followed by placement on the sensor apparatus according to the manufacturer’s instructions. Before each measurement, force and moment readings were zeroed out. The aligner was seated on the sensor apparatus by first seating the anterior teeth and then placing pressure in the posterior direction to fully seat the posterior segments. After insertion, linear forces and moments in the X, Y, and Z planes were simultaneously recorded by force and moment sensors in real time at a sample rate of 600 Hz. The last 8.3 s of stabilized force and moment readings were recorded for statistical analysis. The recorded moment data were mathematically transformed to represent the moments at the point of the approximate center of resistance of the model teeth.

### Statistical analysis

All forces (Fx, Fy, Fz) and moments (Mx, My, Mz) were summarized using medians and interquartile ranges. For each tooth (UR1, UL1, UL2), forces and moments generated by the different tested groups were compared. Kruskal–Wallis test was used for the comparisons using PROC NPAR1WAY with Dwass, Steel, Critchlow–Fligner multiple comparison (post-hoc) tests. All analyses were conducted by using SAS version 9.3 (SAS Inc., Cary, NC). Significance tests were performed by using 2-tailed hypothesis, and the level of significance (α) was set to 0.05. Data were found to be not normally distributed therefore non-parametric comparison tests were employed.

## Results

### Conventions

The resulting forces (Fx, Fy, Fz) and moments (Mx, My, Mz) were recorded in the x, y, and z directions. Fx represents the faciolingual force (+Fx, −Fx), Fy represents the mesiodistal force (+Fy, −Fy), and Fz represents the occlusogingival force (+Fz, −Fz) (Table 1). Moment-to-force ratios in the faciolingual dimension were calculated by the formula My/Fx.

**Table 1:** Multi-axis sensor conventions of direction

Component	Definition	Sign	UL2	UL1	UR1
Fx	Faciolingual	+ −	Lingual Facial	Lingual Facial	Lingual Facial
Fy	Mesiodistal	+ −	Distal Mesial	Distal Mesial	Mesial Distal
Fz	Occlusogingival	+ −	Occlusal Gingival	Occlusal Gingival	Occlusal Gingival
Mx	Angulation	+ −	Mesial Distal	Mesial Distal	Distal Mesial
My	Inclination	+ −	Lingual Facial	Lingual Facial	Lingual Facial
Mz	Rotation	+ −	Mesial Distal	Mesial Distal	Distal Mesial

### Forces and moments on the upper left central incisor

**Fig. 4:**
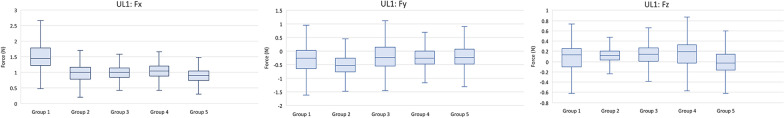
Forces (Fx, Fy, Fz) produced by direct printed aligners on the upper left central incisor

The median stabilized force for all aligners exhibited forces in the palatal direction (Fx). Group 1 active aligners had the highest Fx force levels with a median stabilized force level of 1.44 N, while Groups 2–5 had median stabilized Fx force levels of 0.90–1.01 N. Thus, active DPAs produced forces in the proper direction for the prescribed tooth movement and an increase in labial or lingual thickness causes the aligners to produce optimal forces for bodily translation. For the Fy force levels, all DPAs experienced a slight mesial force. Groups 1, 3, 4, and 5 experienced median mesial forces between 0.23 and 0.27 N, while Group 2 had almost a double median mesial force of 0.51 N. This indicates that Groups 1, 3, 4, and 5 produced the least clinical side effects in the Fy dimension. As for the Fz force levels, all DPAs groups had low levels of extrusive forces that ranged from 0.11–0.19 N, except for Group 5 which experienced less intrusive force of 0.03 N (Fig. 4).

**Fig. 5:**
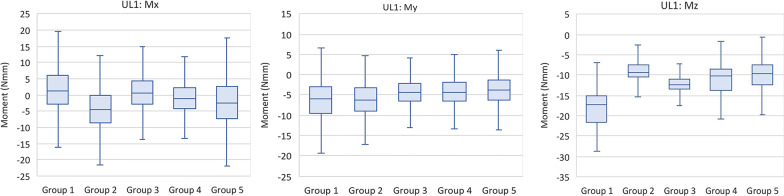
Moments (Mx, My, Mz) generated by direct printed aligners on the upper left central incisor

The median stabilized angulation moments (Mx) were all in the mesial direction, causing the UL1 to tilt toward the midline. Group 1 experienced the greatest Mx moment of 5.97 N mm, while Group 5 experienced the lowest moment of 0.11 N mm. The median stabilized inclination moments (My) were all lingual on the UL1 in the range of 4.95–8.96 N mm. The median stabilized rotation moments (Mz) on the UL1 were all distal-in (mesial-out). Group 1 experienced the most significant distal-in rotational moment of 20.74 N mm, while Group 2 produced the least rotational moment of 11.26 N mm (Fig. 5). Table 2 shows the median and interquartile ranges of forces and moments generated by direct printed aligners of the 5 tested groups on the UL1, while Table 3 demonstrates intergroup comparisons of forces and moments experienced on the UL1.

**Table 2:** Comparison of forces and moments generated by direct printed aligners of the 5 tested groups on the UL1

Force	Group 1		Group 2		Group 3		Group 4		Group 5		*P* value^a^
	Median	IQR	Median	IQR	Median	IQR	Median	IQR	Median	IQR	
Fx	1.44	1.22–1.80	1.00	0.78–1.17	1.01	0.86–1.15	1.04	0.89–1.21	0.90	0.76–1.05	< 0.001
Fy	− 0.27	− 0.64 to 0.03	− 0.51	− 0.75 to − 0.26	− 0.24	− 0.55–0.14	− 0.25	− 0.46 to 0.00	− 0.23	− 0.48 to 0.08	< 0.001
Fz	0.12	− 0.10 to 0.25	0.11	0.03–0.21	0.14	0.01–0.27	0.19	− 0.04–0.33	− 0.03	− 0.17 to 0.14	< 0.001
Mx	5.97	2.29–8.08	0.75	− 0.85 to 3.48	4.06	0.86–5.97	2.02	0.08–4.06	0.11	− 2.74 to 2.56	< 0.001
My	8.96	7.69–11.33	4.95	3.33–6.11	6.10	4.55–8.55	7.35	6.40–8.30	6.16	4.69–7.58	< 0.001
Mz	− 20.74	− 25.71 to − 18.19	− 11.26	− 12.81 to − 9.05	− 14.98	− 16.28 to − 13.17	− 12.67	− 16.57 to − 10.78	− 11.89	− 15.00 to − 9.28	< 0.001

*P* value^a^—overall *p* value for the model

**Table 3:** *P* values for intergroup comparison for forces and moments generated by direct printed aligners on the UL1

UL1 pairwise two-sided multiple comparison analysis						
Dwass, Steel, Critchlow–Fligner Method						
Variable	Fx	Fy	Fz	Mx	My	Mz
Group	*P* value	*P* value	*P* value	*P* value	*P* value	*P* value
1 versus 2	< .0001	< .0001	< .0001	< .0001	< .0001	< .0001
1 versus 3	< .0001	< .0001	< .0001	0.0257	< .0001	< .0001
1 versus 4	< .0001	< .0001	< .0001	< .0001	< .0001	< .0001
1 versus 5	< .0001	< .0001	< .0001	< .0001	< .0001	< .0001
2 versus 3	0.0473	< .0001	< .0001	< .0001	< .0001	< .0001
2 versus 4	< .0001	< .0001	< .0001	< .0001	< .0001	< .0001
2 versus 5	< .0001	< .0001	< .0001	< .0001	< .0001	< .0001
3 versus 4	< .0001	0.0027	< .0001	< .0001	< .0001	< .0001
3 versus 5	< .0001	< .0001	< .0001	< .0001	< .0001	< .0001
4 versus 5	< .0001	< .0001	< .0001	< .0001	< .0001	< .0001

*P* value—Individual groupwise comparisons

### Forces and moments on the upper right central incisor

**Fig. 6:**
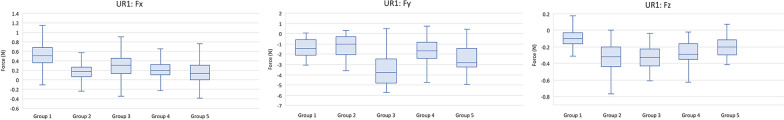
Forces (Fx, Fy, Fz) produced by direct printed aligners on the upper right central incisor

The Fx forces were all directed in the palatal direction for all DPAs. Groups 1 and 3 aligners had median stabilized Fx force levels of 0.50 N and 0.31 N respectively, while Groups 2, 4, and 5 experienced noticeably less Fx force levels from 0.13–0.20 N. The lower Fx forces for Groups 2, 4, and 5 indicate less faciolingual side effects which is favorable. The Fy force levels for all groups were in the distal direction for all aligners. The DPAs ranged in median stabilized force levels of 0.98–3.80 N. Groups 1, 2, and 4 had the least force magnitudes ranging from 0.98 N to 1.67 N. The median stabilized Fz force levels were all intrusive for all the active DPAs. Group 1 was mildly intrusive with a median stabilized force level of 0.10 N, while Groups 2–5 ranged from 0.21 to 0.33 N (Fig. 6).

**Fig. 7:**
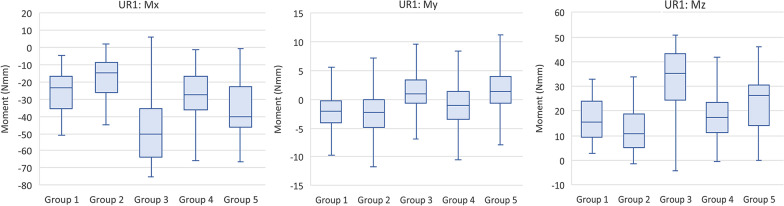
Moments (Mx, My, Mz) generated by direct printed aligners on the upper right central incisor

The median stabilized angulation moments for all groups caused the UR1 to experience a tendency to tip mesially. Group 2 produced the lowest moment of 4.51 N mm, while Groups 1 and 3 produced the most significant moments of more than the double (10.56 N mm and 11.11 N mm respectively). The median stabilized inclination moments were almost all lingual on the UR1 except for Group 2 which had a slight facial moment of 0.72 N mm. Group 4 experienced the lowest lingual moment of 0.46 N mm. Groups 1 and 3 had the most significant lingual moments of 3.30 N mm and 4.24 N mm respectively. The median stabilized rotation moments (Mz) on the UR1 were all distal-in (mesial-out). Groups 3 and 5 experienced the most significant rotational moments of 45.64 N mm and 33.76 N mm respectively, while Group 2 produced the lowest moment of 13.29 N mm (Fig. 7). Table 4 shows the median and interquartile ranges of forces and moments generated by direct printed aligners of the 5 tested groups on the UR1, while Table 5 demonstrates intergroup comparisons of forces and moments experienced on the UR1.

**Table 4:** Comparison of forces and moments generated by direct printed aligners of the 5 tested groups on the UR1

Force	Group 1		Group 2		Group 3		Group 4		Group 5		*P* value^a^
	Median	IQR	Median	IQR	Median	IQR	Median	IQR	Median	IQR	
Fx	0.50	0.36–0.68	0.17	0.07–0.27	0.31	0.13–0.46	0.20	0.10–0.32	0.13	− 0.01 to 0.30	< 0.001
Fy	− 1.41	− 2.08 to − 0.60	− 0.98	− 2.05 to − 0.30	− 3.80	− 4.79 to − 2.47	− 1.67	− 2.41 to − 0.85	− 2.82	− 3.22 to − 1.43	< 0.001
Fz	− 0.10	− 0.16 to − 0.03	− 0.32	− 0.44 to − 0.20	− 0.33	− 0.43 to − 0.23	− 0.28	− 0.35 to − 0.16	− 0.21	− 0.30 to − 0.12	< 0.001
Mx	− 10.56	− 13.01 to − 8.64	− 4.51	− 6.71 to − 2.93	− 11.11	− 11.85 to − 8.71	− 9.46	− 10.55 to − 8.39	− 8.95	− 10.88 to − 7.25	< 0.001
My	3.30	2.12–4.27	− 0.72	− 2.44 to 0.88	4.24	1.99–5.32	0.46	− 0.94 to 2.01	2.58	1.19–4.25	< 0.001
Mz	18.78	10.36–29.20	13.29	5.91–24.62	45.64	30.85–56.10	21.78	13.29–29.89	33.76	17.55–38.91	< 0.001

*P* value^a^—overall *P* value for the model

**Table 5:** *P* values for intergroup comparison for forces and moments generated by direct printed aligners on the UR1

UR1 Pairwise Two-Sided Multiple Comparison Analysis						
Dwass, Steel, Critchlow–Fligner Method						
Variable	Fx	Fy	Fz	Mx	My	Mz
Group	*P* value	*P* value	*P* value	*P* value	*P* value	*P* value
1 versus 2	< .0001	< .0001	< .0001	< .0001	< .0001	< .0001
1 versus 3	< .0001	< .0001	< .0001	< .0001	< .0001	< .0001
1 versus 4	< .0001	< .0001	< .0001	< .0001	< .0001	< .0001
1 versus 5	< .0001	< .0001	< .0001	< .0001	< .0001	< .0001
2 versus 3	< .0001	< .0001	< .0001	< .0001	< .0001	< .0001
2 versus 4	< .0001	< .0001	< .0001	< .0001	< .0001	< .0001
2 versus 5	< .0001	< .0001	< .0001	< .0001	< .0001	< .0001
3 versus 4	< .0001	< .0001	< .0001	< .0001	< .0001	< .0001
3 versus 5	< .0001	< .0001	< .0001	< .0001	< .0001	< .0001
4 versus 5	< .0001	< .0001	< .0001	< .0001	< .0001	< .0001

*P* value—Individual groupwise comparisons

### Forces and moments on the upper left lateral incisor

**Fig. 8:**
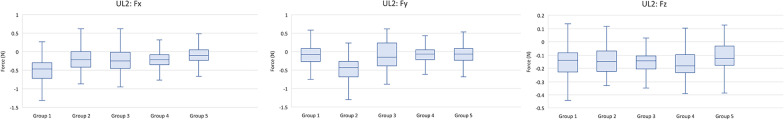
Forces (Fx, Fy, Fz) produced by direct printed aligners on the upper left lateral incisor

The Fx force levels for all groups were in the facial direction for all aligners. Group 1 DPAs exhibited a median stabilized force level of 0.47 N, while Groups 2–5 exhibited less median stabilized force levels from 0.09 to 0.25 N. This indicates that increased labial or lingual aligner thickness reduced the Fx side effect forces on the UL2. The Fy force levels for all DPA groups were in the mesial direction. The median stabilized force levels of Groups 1, 3, 4, and 5 ranged from 0.07 to 0.15 N, while Group 2 experienced a higher stabilized median force level of 0.42 N. The Fz force levels were all slightly intrusive for all DPA groups. Aligners experienced a median stabilized intrusive force levels of 0.13–0.18 N (Fig. 8).

**Fig. 9:**
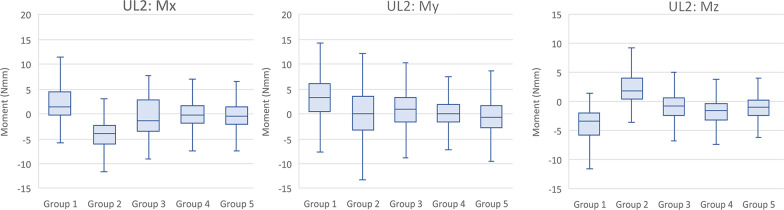
Moments (Mx, My, Mz) generated by direct printed aligners on the upper left lateral incisor

The median stabilized angulation moments for essentially all groups caused the UL2 to experience a tendency to tip mesially. Group 2 produced distal tip but at a low magnitude of 0.01 N mm, virtually no side effects. Groups 3, 4, and 5 generated moments of about 0.30 N mm to 0.63 N mm, while Group 1 produced the most significant moment of about 2.6 N mm. This is another indication that increased labial or lingual thicknesses reduced angulation (Mx) moments side effects. The median stabilized inclination moments (My) were all facial on the UL2 in the range of 1.63–2.79 N mm. These moment values are approximately one-third the magnitude of the My moments experienced by the UL1. The median stabilized rotation moments (Mz) on the UL2 were variable and random in their rotational direction. Groups 1 and 2 experienced the most significant moment magnitudes of 2.43 N mm and 3.26 N mm, respectively. Groups 3, 4, and 5 had the least Mz side effect moments ranging in magnitude from 0.75 to 1.07 N mm (Fig. 9). Table 6 shows the median and interquartile ranges of forces and moments generated by direct printed aligners of the 5 tested groups on the UL2, while Table 7 demonstrates intergroup comparisons of forces and moments experienced on the UL2.

**Table 6:** Comparison of forces and moments generated by direct printed aligners of the 5 tested groups on the UL2

Force	Group 1		Group 2		Group 3		Group 4		Group 5		*P* value^a^
	Median	IQR	Median	IQR	Median	IQR	Median	IQR	Median	IQR	
Fx	− 0.47	− 0.71 to − 0.30	− 0.21	− 0.41 to 0.00	− 0.25	− 0.44 to − 0.02	− 0.20	− 0.35 to − 0.08	− 0.09	− 0.23 to 0.06	< 0.001
Fy	− 0.08	− 0.26 to 0.10	− 0.42	− 0.68 to − 0.27	− 0.15	− 0.39 to 0.23	− 0.07	− 0.22 to 0.05	− 0.07	− 0.22 to 0.09	< 0.001
Fz	− 0.14	− 0.23 to − 0.08	− 0.15	− 0.22 to − 0.07	− 0.14	− 0.21 to − 0.11	− 0.18	− 0.23 to − 0.10	− 0.13	− 0.18 to − 0.03	< 0.001
Mx	2.60	2.03–3.45	− 0.01	− 0.37 to 0.32	0.39	− 0.05 to 0.85	0.63	0.05–1.22	0.30	− 0.14 to 0.71	< 0.001
My	− 2.64	− 3.83 to − 1.65	− 2.79	− 3.70 to − 1.81	− 2.07	− 3.45 to − 0.78	− 2.53	− 3.29 to − 1.82	− 1.63	− 2.57 to − 0.79	< 0.001
Mz	− 2.43	− 5.59 to − 0.83	3.26	1.45–5.96	0.10	− 2.66 to 2.08	− 1.07	− 2.98 to 0.48	− 0.75	− 2.36 to 0.94	< 0.001

*P* value^a^—overall *P* value for the model

**Table 7:** *P* values for intergroup comparison for forces and moments generated by direct printed aligners on the UL2

UL2 Pairwise Two-Sided Multiple Comparison Analysis						
Dwass, Steel, Critchlow–Fligner Method						
Variable	Fx	Fy	Fz	Mx	My	Mz
Group	*P* value	*P* value	*P* value	*P* value	*P* value	*P* value
1 versus 2	< .0001	< .0001	< .0001	< .0001	0.9551*	< .0001
1 versus 3	< .0001	< .0001	< .0001	< .0001	< .0001	< .0001
1 versus 4	< .0001	0.3299*	< .0001	< .0001	< .0001	< .0001
1 versus 5	< .0001	< .0001	< .0001	< .0001	< .0001	< .0001
2 versus 3	< .0001	< .0001	< .0001	< .0001	< .0001	< .0001
2 versus 4	0.4218*	< .0001	< .0001	< .0001	< .0001	< .0001
2 versus 5	< .0001	< .0001	< .0001	< .0001	< .0001	< .0001
3 versus 4	< .0001	< .0001	< .0001	< .0001	< .0001	< .0001
3 versus 5	< .0001	< .0001	< .0001	< .0001	< .0001	< .0001
4 versus 5	< .0001	< .0001	< .0001	0.0056	< .0001	0.0041

*P* value—Individual groupwise comparisons

### Moment-to-force ratios

The moment-to-force ratios were calculated for each group of DPAs as shown in Table 8**.** The DPAs largely produced controlled tipping on the UL1 and UR1 teeth. On the UL2, the DPAs produced mostly root movement except for Groups 1 and 3 which experienced controlled tipping and uncontrolled tipping, respectively. Group 3 DPAs was the only group that did not produce controlled tipping on the UL1 which had the digitally programmed bodily movement.

**Table 8:** Faciolingual moment-to-force ratios experienced by the five tested groups of direct printed aligners for all teeth (My/Fx)

Tooth	Group 1	Group 2	Group 3	Group 4	Group 5
UR1	6.60	4.24	2.62	2.30	19.85
UL1	6.22	4.95	0.67	7.07	6.84
UL2	5.62	13.29	0.19	12.65	18.11

**Fig. 10:**
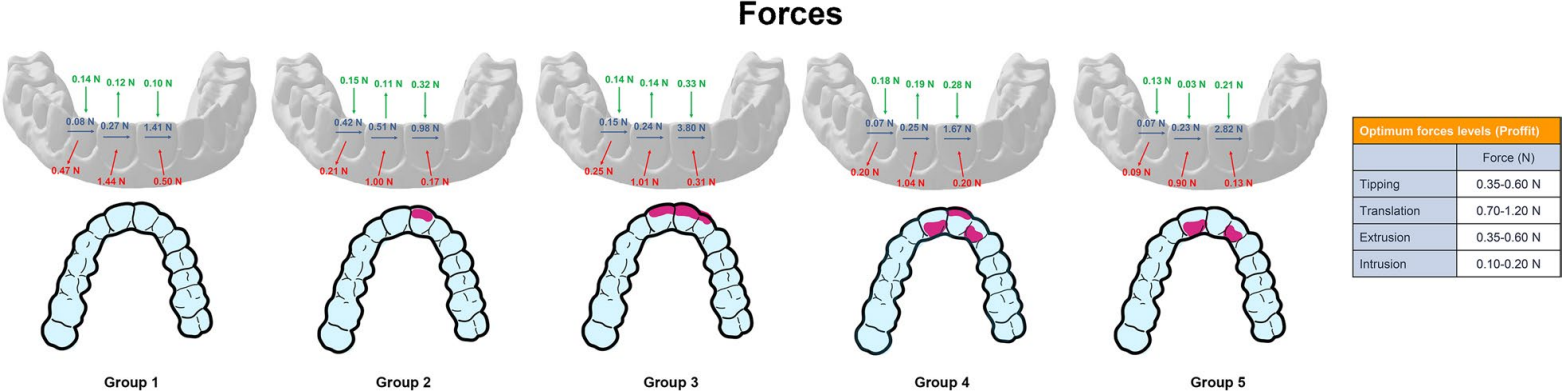
Comparison of forces produced by direct printed aligners on different teeth between the five tested study groups

**Fig. 11:**
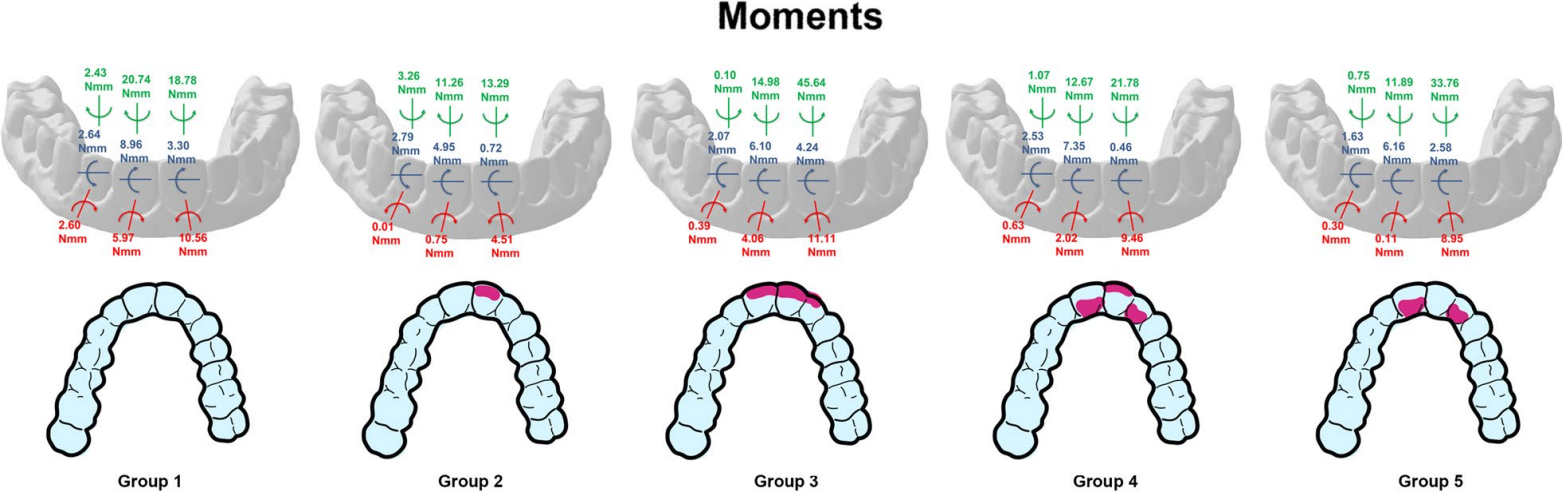
Comparison of moments generated by direct printed aligners on different teeth between the five tested study groups

All the findings between groups were significant except for some readings on the UL2, where Groups 2 and 4 were not significantly different for Fx forces; Groups 1 and 4 were not significantly different for Fy forces; and Groups 1 and 2 were not significantly different for My moments. A summary of forces and moments generated by the DPAs is illustrated in Figs. 10 and 11 for all tested teeth.

## Discussion

The direct printing of clear aligners has recently been introduced to increase manufacturing precision and reduce errors, ultimately achieving greater predictability of tooth movements. TC-85, a clear and biocompatible photocurable resin with favorable mechanical properties for clear aligner therapy, including elasticity, strength and a unique shape memory effect, has become available on the market. One of the significant advantages of directly printed aligners is the design flexibility, including the ability to change material thickness and control the offset between the aligner and the dentition, resulting in a firmer grip and better tracking of programmed movements [20]. From a biomechanical perspective, the direction and amount of tooth movement should be combined with material geometry considerations, such as material thickness, for optimal treatment efficiency with clear aligners. Therefore, the present study utilized an approach that directly measures moments and forces in all three planes of space in real time for three adjacent teeth during lingual movement of a single tooth.

Previous research has suggested that the ideal aligner activation increment should range from 0.20 to 0.50 mm [21]. Additionally, research by Hertan et al. reported that direct 3D-printed aligners generate significantly less force than conventional thermoformed aligners [22]. Therefore, the present study decided to test the activation of a 0.50 mm increment for DPAs, corresponding to the higher end of the recommended activation increments for thermoformed aligners.

A significant advantage of the experimental setup presented in this study is that it tests the aligners at intra-oral temperatures. This is crucial since the Tera Harz resin used to fabricate the DPAs has a shape memory effect that is temperature-dependent. Maintaining an experimental apparatus at a constant intra-oral temperature of 37 °C should result in more accurate recordings of forces and moments generated by the DPAs than if the experiments were run at room or uncontrolled temperature. Another reason for simulating intra-oral conditions regarding temperature is to provide a more meaningful interpretation of in vitro experimental results.

### Effects of increased surface thickness in the Fx dimension

Palatal forces were experienced by UL1 and UR1 for all aligner groups, while UL2 experienced facial forces regardless of the aligner group. Analysis of the data showed that a general pattern emerged where increased facial or lingual DPA surface thickness resulted in lower force levels in the Fx dimension, the primary dimension of the programmed lingual tooth movement. This finding is opposite to thermoformed aligners, where previous research has stated that increased thickness of thermoformed aligners results in greater forces generated [10, 13]. However, previous research tested conventional aligners where the entire aligner is thicker rather than selected locations only.

The Fx forces experienced by the UL1 tooth in baseline Group 1 were 1.44 N, while Groups 2, 3, and 4 with increased facial thicknesses exhibited force levels between 1.00 and 1.04 N. Group 5 had increased lingual thickness and had a slightly lower Fx magnitude of 0.90 N. This corresponds to approximately a 30% decrease in force magnitude in the Fx dimension experienced by the UL1 during lingual movement due to increased facial or lingual thickness. Proffit states that the ideal force magnitudes for bodily movement are 70–120 g [16]. Groups 2–4 with increased facial or lingual thickness generated ideal Fx forces for bodily movement, while Group 1 with uniform thickness generated higher than ideal force magnitudes.

This pattern is also true for the aligners on the UR1 and UL2 teeth, where DPAs for Groups 2–5 generated 22–81% less Fx force magnitude than their respective Group 1 baselines. Hence, it can be noted that, in most cases, the increased labial or lingual aligner thickness appeared to reduce the magnitude of Fx side effect forces on adjacent teeth.

### Effects of increased surface thickness in the Fy dimension

The DPAs demonstrated an unexpected force profile in the Fy direction. Regardless of aligner group, the UR1 experienced distal forces, while the UL1 and UL2 experienced mesial forces. This resulted in a midline shift toward the right as the DPAs attempted to express the lingual movement of the UL1.

According to Proffit, the optimal tipping force magnitude is 35–60 g [16]. Groups 1, 3, 4, and 5 generated Fy forces of approximately 15 g or less on the UL2, and Fy forces of approximately 27 g or less on the UL1. In contrast, Group 2 generated around 42 and 51 g of force on the UL2 and UL1, respectively. As a result, Group 2 DPAs generated unintended Fy forces within the optimal tipping force ranges, while the other groups generated forces below the mentioned range. Therefore, it can be stated that Groups 1, 3, 4, and 5 DPAs generated the least side effects in the mesiodistal direction.

The DPAs generated unusually high force levels on the UR1 ranging from 0.98–3.80 N in the distal direction. Group 2 generated the lowest distal force of 98 g, while Group 1 and Group 4 aligners generated the next lowest force levels of 141 g and 167 g, respectively. Group 3 generated the highest distal force level of 380 g. Groups 1, 2, and 4 DPAs generated the least side effects in the mesiodistal direction.

### Effects of increased surface thickness in the Fz dimension

The UR1 and UL2 experienced intrusion, while the UL1 experienced extrusive forces as it moved lingually. This was expected due to the relative extrusion that is typically observed when moving the UL1 lingually, and the intrusive forces experienced by the adjacent teeth that provide anchorage for this movement. The UR1 experienced intrusive force magnitudes in the range of 10–33 g, while the UL2 experienced intrusive forces in the range of 13–18 g. On the other hand, the UL1 experienced extrusive force magnitudes in the range of 11–19 g, with an unexpected intrusive force of 3 g for Group 5. Proffit recommends optimal intrusive force magnitudes of 10–20 g, while the optimal extrusive force magnitudes are 35–60 g.^16^ Therefore, the extrusive force side effects experienced by the UL1 are minimal, while the UR1 and UL2 experience enough force to produce clinical side effects. Group 1 and Group 5 aligners generated the least amount of intrusive side effects on the UR1 and UL2.

### Effect of moments generated by direct printed aligners

The UL1 and UR1 experienced the same type of moments with mesial tipping in the Mx dimension, lingual moments in the My dimension, and distal-in moments in the Mz dimension. This pattern was the same for the UL2, only in the Mx dimension, but was opposite for the My dimension where it experienced facial moments. Moreover, the Mz dimension showed variable and random patterns of expressing rotations. Based on the findings of the present study, clear aligners can produce unpredictable forces and moments measured on adjacent teeth, highlighting the complex nature of clear aligner biomechanics, and may help explain the lower clinical predictability with this treatment modality.

In traditional biomechanics for fixed appliances, a lingually directed force on the central incisor would result in a reciprocal force in the facial direction on the adjacent teeth. Nevertheless, this is not always the case with clear aligners. These findings contradict with the findings by Kaur et al. [23] using a mechanical simulator to measure forces and moments generated on teeth for 0.20 mm buccal movement using clear aligners. They found that for the maxillary right central incisor, a buccal force produced a facial crown-tipping moment; clinically relevant reciprocal forces were seen on adjacent teeth, with lingual forces on both adjacent teeth, and a lingual crown-tipping moment on the adjacent central incisor, but no clinically significant moment on the lateral incisor.

The best aligner would theoretically: (i) produce ideal force magnitudes in the direction of the programmed tooth movement, (ii) produce an ideal force-to-moment ratio for the prescribed type of tooth movement, (iii) produce the least amount of side effects on the tooth with programmed movement in the orthogonal dimensions, and (iv) produce the least amount of side effects on the adjacent teeth in all dimensions. Creating a force system for achieving bodily movement with clear aligners is extremely challenging, especially without auxiliary features such as attachments and pressure points [12, 24]. This is because effective torque control is contingent on the creation of a counter-moment to oppose the moment of force which initially acts on the tooth. In clear aligner therapy, this is achieved with an intimate fit of the aligner with the tooth; the initial tipping of the tooth and reversible deformation of the aligner create new contact points which generate a moment of couple. With a suboptimal aligner fit, this counter-moment cannot be produced, resulting in torque loss.

From the perspective of linear forces, Groups 2–5 generate the most ideal Fx force magnitudes for translating teeth, with Group 4 aligners obviously generating the least amount of side effects across all teeth, and Group 1 generating higher than ideal forces in the Fx dimension. In the Fy dimension, increased DPA thickness does not significantly increase side effects. For the Fz dimension, adding additional thickness does not appear to confer significant side effects. The force levels remained relatively constant except for a slight increase in intrusive forces on the UR1 from increased aligner thickness.

From the perspective of moment-to-force ratios, Groups 4 and 5 produce the ratios closest to true bodily translation on the UL1. However, Group 5 produced root movement moment-to-force ratios on the adjacent teeth. Group 1 produced controlled tipping on all teeth involved. Group 3 produced the worst moment-to-force ratio producing uncontrolled tipping for the UL1. Thus, increasing facial thickness across multiple teeth may lead to DPAs producing unfavorable moment-to-force ratios, while adding thickness to the lingual surface of adjacent teeth may improve moment-to-force ratios on the tooth when programmed with lingual translation. These findings are consistent with previous studies by Hahn et al. [12] and ElKholy et al. [24] who demonstrated that effective incisor torque could not be achieved during bodily movement with aligners.

Taking all of these factors into consideration, Group 4 best optimized the force level and moment-to-force ratio for programmed tooth movement and minimized force and moment side effects to a reasonable degree. Therefore, increased aligner thickness on the surface that trails the programmed tooth movement, combined with increased thickness on the opposite aligner side on the adjacent teeth that serve as anchorage units, seems to produce advantageous biomechanics for direct 3D-printed aligners. Hypothetically, it can be stated that an aligner that is selectively thicker where more stabilization or less tooth movement is desired and thinner where tooth movement is desired, at least on the surface that trails tooth movement, may also confer advantageous clinical properties. Nonetheless, the general finding still shows that the presence of increased DPA thickness around the programmed tooth movement reduces the overall force level and side effects compared to a thinner aligner.

While fixed appliance biomechanics are dictated by a single point of contact between arch wires and bracket slots, clear aligners contact the entire crown surfaces as well as varying tooth surfaces at different stages of treatment. This greatly complicates the biomechanics of clear aligners, since the force systems can no longer be reduced to mathematical vectors as easily. Conceptually, clear aligners biomechanics cannot be reduced to simple actions and counteractions as observed with fixed appliances either.

It is important to note that the current study has certain limitations. Firstly, it was an in vitro benchtop study, which means that it was not conducted in a natural environment and did not account for the natural space and elastic behavior of periodontal ligaments (PDL). In humans, the average width of PDL is between 0.15 and 0.38 mm, and it may widen during orthodontic tooth movement. The PDL acts as a shock absorber, compressing and dampening the moments and forces experienced by teeth [25]. The in vitro array of sensors used in the study did not take into account the buffering nature of the PDL space. Furthermore, the study did not simulate the effects of masticatory occlusal forces [26] or the impact of saliva [27] on the performance of clear aligners. These factors should be considered in future studies to provide a more accurate understanding of the biomechanics of clear aligners.

## Conclusions

The thickness and surface geometry of direct 3D-printed aligners can be altered to optimize the magnitudes of forces and moments generated by the aligners, while minimizing side effects. Increasing the aligner thickness on the surface that trails the programmed tooth movement, along with increasing thickness on the opposite aligner side on the adjacent teeth that serve as anchorage units, has been found to produce advantageous biomechanics for direct 3D-printed aligners. Overall, the presence of increased aligner thickness around the programmed tooth movement reduces the overall force level and side effects compared to a thinner aligner.

## Data Availability

The datasets used and/or analyzed during the current study are available in the paper.
